# A Simplified Protocol for Reversing Phenotypic Conversion of *Ralstonia solanacearum* during Experimentation

**DOI:** 10.3390/ijerph17124274

**Published:** 2020-06-15

**Authors:** Pramod Kumar Sahu, Shailendra Singh, Amrita Gupta, Udai B. Singh, Surinder Paul, Diby Paul, Pandiyan Kuppusamy, Harsh V. Singh, Anil Kumar Saxena

**Affiliations:** 1 ICAR-National Bureau of Agriculturally Important Microorganisms, Maunath Bhanjan UP-275103, India; singh.shailendra512@gmail.com (S.S.); amritasoni90@gmail.com (A.G.); udaiars.nbaim@gmail.com (U.B.S.); surinderpaulsandhu@gmail.com (S.P.); drharsh2006@rediffmail.com (H.V.S.); saxena461@yahoo.com (A.K.S.); 2 Pilgram Marpeck School of Science, Technology, Engineering and Mathematics, Truett McConnel University, 100 Alumni Dr. Cleveland, GA 30528, USA; dpaul@truett.edu; 3 ICAR-Central Institute for Research on Cotton Technology, Ginning Training Centre, Nagpur, Maharashtra 440023, India; pandiannkl@gmail.com

**Keywords:** *Ralstonia solanacearum*, virulence, virulence induction protocol, *Phyllanthus emblica*, nutrient deprivation

## Abstract

Background: *Ralstonia solanacearum* has the problem of losing the virulence in laboratory conditions, during prolonged experimentation. Since pure colonies of *R. solanacearum* contain cell fractions differing in virulence, it was considered worthwhile to find a way of selecting the cells with lower attenuation. Therefore, a methodology for inducing virulent-type colonies occurrence in *Ralstonia solanacearum* was developed. Methods: Nutrient gradient was created by swabbing *R. solanacearum* culture in a slanted KMTTC medium, and *Phyllanthus emblica* extract was given by well diffusion. Live–dead cell imaging using *Bac*Light, effects of ascorbic acid on cell viability, and production of virulence factors (exopolysaccharides, cellulase, and pectinase) supported this hypothesis. The tagging of *R. solanacearum* with green fluorescent protein and further confocal scanning laser microscopic visualization confirmed the colonization in vascular bundles of tomato. Results: *P. emblica* extract suppressed *R. solanacearum* initially in well diffusion, but further developed virulent-type colonies around the wells. Nutrient deprivation was found to have synergistic effects with *P. emblica* extract. The converted fluidal (virulent type) colonies could be able to colonize vascular bundles and cause wilting symptoms. Conclusion: This method will be useful in the laboratories working on biocontrol of *R. solanacearum* for maintaining virulent-type colonies. Moreover, it could form the basis for studies on the stability of phenotypic conversion and cell fractions in *R. solanacearum*.

## 1. Introduction

*Ralstonia solanacearum* is a devastating pathogen-causing bacterial wilt in over 450 plant species [1]. The catastrophic nature of this pathogen has made it the second most economically important bacterial pathogen of plants [2]. It is a pathogen of the vascular system that invades root xylem vessels and spreads into the stem tissue, where it produces excessive exopolysaccharides (EPSs). The EPSs clog the xylem vessels and cause wilting in the host plant. The *R. solanacearum* can respond to the changes in the environment by changing the physiological state from virulent to avirulent, and vice versa [3], termed as phenotypic conversion (PC). Due to this, many of the *R. solanacearum* strains quickly lose their virulence during subculturing [4]. This sudden shift in colony morphology renders it non-fluidal and impaired in causing disease [5]. It is problematic for proving pathogenicity in general, and biocontrol studies in particular [6]. The research on *R. solanacearum* is more than a century old. In the review “One Hundred and One Years of Research on Bacterial Wilt” by Kelman [7], the rapid loss of virulence in *R. solanacearum* was indicated as a significant obstacle. This issue was recognized by Smith [8], who stated the species had “more variation in virulence and more failure on the way of inoculation as compared to almost all other species”. One of the possible reasons behind this PC is the presence of different cell fractions in a single (so-called) pure culture [9,10]. Studies using ion-exchange chromatography indicated that there are three individual fractions in a pure culture of *R. solanacearum* [9,10]. These cell fractions had a different attenuation index (attenuation index calculated by the diameter of the red spot in colony/total colony diameter) and formed colonies with different pathogenicity under varying culture conditions. Rapid conversion of colony physiology renders it unsuitable for uniform experimentation in disease development studies as it becomes avirulent. These facts signify that the variation in virulence is one of the severe concerns working with this pathogen.

Studies on the relationship between colony morphotypes and virulence showed that fluidal colonies with a white-to-pink center are virulent, whereas, deep red, butyrous circular colonies with a narrow blue border cause little or no damage to plants [11]. EPS is a heterogeneous polymer of *N*-acetylated EPS I and a key virulence factor of *R. solanacearum*. PC of wild-type *R. solanacearum* from fluidal white to non-fluidal red colony renders it avirulent or weakly virulent [4,12,13,14]. Previous studies have shown that phenotypically converted strains when applied along with the wild type suppressed pathogenesis caused by wild-type strains and conferred no wilting [4,5,15]. Colonization of eggplant by avirulent PC strains could protect the plant from bacterial wilt [16]. However, information on further physiological changes in PC strains (reversal of virulence/occurrence of fluidal colonies) was lacking, and thus the stability of these PC strains is required to be studied. Since there were reports that *R. solanacearum* is a mixture of different cell fractions varying in degrees of attenuation [9,10], there is a spontaneous occurrence of few fluidal colony types in PC strains. However, the frequency is shallow and unpredictable. Studies on PC is not only significant for maintaining virulent colony types for laboratory studies, but also for the stability of avirulent strain applied for cross-protection. Although the virulence is controlled by a very complex fine-tuned system, including five genes *phc* system as reviewed by Schell [17], some of the external factors could also influence virulence in *R. solanacearum*. Since bioactive roles of different plant extracts against *R. solanacearum* were reported [18,19], our initial screening indicated inductive effects of *Phyllanthus emblica* on pure colonies. Along with *P. emblica* extract, we also found that nutrient deprivation could also selectively activate virulent-type fractions from pure colonies of PC strains. Therefore, in the present study, a protocol for producing virulent colony types from PC strains in *R. solanacearum* was developed.

## 2. Material and Methods

### 2.1. Ralstonia solanacearum Strains

**Figure 1:**
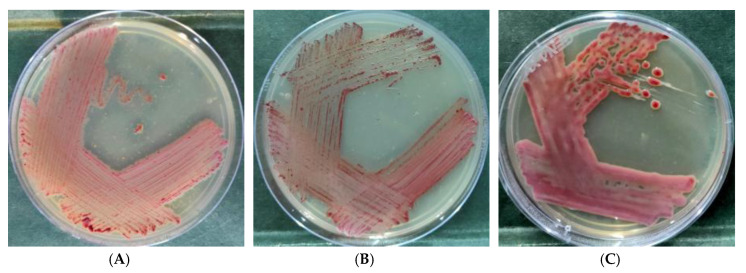
Colony morphology of *R. solanacearum* NAIMCC-B-01630 used in the study: (**A**) non-fluidal, (**B**) butyrous, and (**C**) fluidal colonies.

Two *R. solanacearum* cultures used in this study were taken from National Agriculturally Important Microorganisms Culture Collection (NAIMCC), Indian Council of Agricultural Research- National Bureau of Agriculturally Important Microorganisms, Mau with accession numbers NAIMCC-B-01630 and TB-01838. The *R. solanacearum* cultures used in the present study were acquired in the year 2015 and maintained in glycerol stock, slants, and active plates in the lab. These experiments have been conducted since the year 2015 and were repeated ten times with 15 different strains of *Ralstonia* for validity and stability of the hypothesis. These cultures were grown in KMTTC medium (casein hydrolysate 1 g/L, peptone 10 g/L, glucose 5 g/L, and agar-agar 15 g/L supplemented with 2,3,5 Triphenyl tetrazolium chloride at 0.005%) [11]. *R. solanacearum* NAIMCC-B-01630 was isolated from eggplant (phylotype I), and TB-01838 was isolated from chili (phylotype I). The pathogenicity of these strains on tomato was confirmed. The variations of the colonies (butyrous, fluidal, and non-fluidal; Figure 1) were collected from spontaneously generated phenotypic conversion colonies from several plates and used for this study. The term butyrous was used for PC strains with an intermediate colony texture (semi-fluidal, pale-to-dark red, and flat surface) to fluidal and non-fluidal colonies.

### 2.2. PC Reversal and Virulence Induction Protocol

#### 2.2.1. Preparation of *P. emblica* Extract

Clean and bruise-free fruits of *P. emblica* (Indian gooseberry) were taken for the study. These fruits were crushed with a mortar and pestle, after removing seeds. The fruit pulp weighing about 30 g was further crushed with a clean pestle and mortar, using 100 mL of phosphate buffer saline (1X = 137 mM NaCl, 10 mM phosphate, 2.7 mM KCl). The entire content was filter-sterilized twice, using a 0.22 µm syringe filter. The extract was preserved at 4 °C, till use.

#### 2.2.2. Preparation of Nutrient Gradient and Inoculation

Gradient for sequential nutrient deprivation was created in the plate by pouring agar medium in a slanting manner. In the first study, a full-strength KMTTC medium was used, whereas, in the second study, 1/50th concentration was used. In both the studies, a gradient of nutrients was created by pouring approximately 40 mL of medium to a square Petri plate (120 × 120 mm). One side of the plate was kept on higher elevation to get a uniform slant till the center of plates. The plates were inoculated by swabbing 0.2 OD (at 600 nm) two-day-old *R. solanacearum* culture onto the slanting surface. After drying of inocula for 3 h, agar wells of 6 mm diameter were filled with *P. emblica* extract (100 µL, filter-sterilized). The plates were incubated at 28 ± 2 °C for 3 days, and its effect on the appearance of virulent type (fluidal) colonies was observed.

### 2.3. Effect of P. emblica Extract and Nutrient Deprivation on PC Reversal

#### 2.3.1. Live–Dead Cell Imaging

LIVE/DEAD^®^ *Bac*Light™ bacterial viability staining was done for differentiating live and dead bacterial cells around the agar wells (from the Section 2.2.2). *Bac*Light contains SYTO-9 and propidium iodide (PI) in equal proportions. SYTO-9 stains all the bacterial cells and emits green–yellow fluorescence, and PI stains cells with compromised membranes (i.e., dead cells) and emits red fluorescence. In this study, one sample was taken from the region near the agar well (designated as “a”; region inside 14 mm diameter of suppression zone), and another from the zone away to the agar well (“b”; region outside 14 mm diameter of suppression zone). Both the samples were stained by using *Bac*Light and visualized under a confocal scanning laser microscope (CSLM; Nikon Eclipse 90i, Minato City, Tokyo, Japan). Two laser sources (488 and 543 nm) were used to excite the sample, and images were acquired by using the NIS element 3.2.3 program (Nikon).

#### 2.3.2. Bacteriostatic Effect of Ascorbic Acid Present in *P. emblica* Extract

The impact of Indian gooseberry extract on pH of the medium was tested. The acidic range filter paper (Himedia) was used on diffusion well and surrounding zone after 1 h of diffusion. Since the reduction of pH was observed around the well, and Indian gooseberry is rich in ascorbic acid, the effect of ascorbic acid on cell viability of *R. solanacearum* NAIMCC-B-01630 was assessed. In this experiment, 10,000 ppm ascorbic acid was used to accord the ascorbic acid content of *P. emblica* fruit [20]. A duplicate set was kept with *P. emblica* extract for comparison. Growth in 24 h was measured to understand the decline in cell viability with time. In an Eppendorf Tube, 24-hour-old culture in CPG broth (casamino acid 1.0 g/L, peptone 10 g/L, glucose 5 g/L) was taken. A series of 0, 20, 40, 60, 80, 100, 120, 140, 160, 180, and 200 µL of ascorbic acid or *P. emblica* extract was added to make a final volume of 200 µL with 24-hour-old broth. In the case of a 200 µL vial, 20 µL of inoculum was added separately. Viable cell count was assessed after 12 h, by serial dilution plate count method.

#### 2.3.3. Effect on Other Virulence Factors

The effect of this treatment on two other virulence factors, viz. cellulase and pectinase production, was tested. Colonies from “a” and “b” zones (as explained in the Section 2.3.1) were compared for production of cellulase and pectinase enzymes. The qualitative estimation was done, and the size of the zone was compared. Pectinase production by *R. solanacearum* NAIMCC-B-01630 culture was compared as described by Durairaj et al. [21]. Briefly, the pectin-supplemented minimal medium was inoculated with 24-hour-old *R. solanacearum* culture, followed by incubation at 28 ± 2 °C for 72–96 h. Post-incubation, plates were flooded with 0.1% Congo red solution for 20 min, followed by washing with 1M NaCl for 15 min. The appearance of the orange clearing zone around the bacterial colonies indicated pectinase activity. Similar to pectinase production, the minimal medium was supplemented with 1% CMC (carboxy methyl cellulose) for cellulase production. These plates were inoculated with 24-hour-old *R. solanacearum* culture and incubated for a period of 48 h, at 28 ± 2 °C. After incubation, the plates were flooded with 0.1% Congo red for 20 min, followed by washing with 1M NaCl for 15 min. The development of an orange zone around the colonies indicated cellulase activity [22].

#### 2.3.4. *GFP* Tagging of *R. solanacearum* Strains

**Figure 2:**
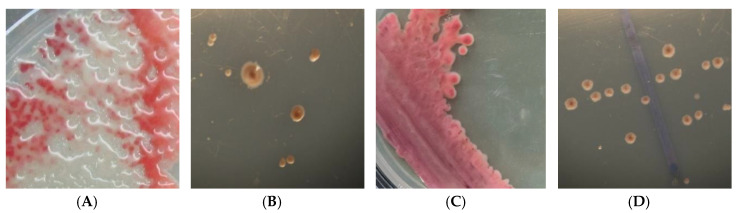
Colony morphology of converted fluidal strain before and after *gfp* labeling: (**A**) colonies before *gfp* labeling in Petri plate, (**B**) single colony morphology in stereo microscope before *gfp* labeling, (**C**) colonies after *gfp* labeling in Petri plate, and (**D**) single colony morphology in stereo microscope after *gfp* labeling.

*R. solanacearum* NAIMCC-B-01630 was tagged with *gfp* by triparental mating with *Escherichia coli* S17-1 containing Tn5 *gus*A *gfp* cassette and *E. coli* HB101 containing pRK2013 [23]. Briefly, transconjugants were selected on Luria Bertani (LB) agar plates containing nalidixic acid and kanamycin. The positive transconjugants were selected by visualizing under UV for fluorescence. The presence of *gfp* in the selected transconjugants was also confirmed by amplifying 650 bp of the amplicon, using YL065 (F) 5′-GCGATGTTAATGGGCAAAAA-3′ and YL066 (R) 5′-TCCATGCCATGTGTAATCCT-3′ as primers. The selected GFP-expressing transconjugants of *R. solanacearum* NAIMCC-B-01630 strains were used to carry out further experiments after confirming that the *gfp* tagging did not affect the pathogenicity of the bacterium. The positive transformants were compared with the wild-type strain to see if there was any change due to *gfp* on colony morphology, growth characteristics, and virulence. The transformants exhibiting behavior similar to the wild type were selected and used in further experiments. The colony morphology of *R. solanacearum* NAIMCC-B-01630 used for tagging was fluidal, as shown in Figure 2.

#### 2.3.5. In Vivo Confirmation of Virulence

Three-week-old tomato seedlings of two varieties, viz. Arka Rakshak (AR; multiple disease tolerant hybrid) and Pusa Ruby (PR; susceptible check), grown in sterilized soil, were used for testing the virulence of *R. solanacearum*. Inoculation of 1 mL 0.2 OD (at 600 nm) 24-hour-old culture was done in the root zone of the plants grown in 96-well protrays. Fluidal and non-fluidal types of colonies were compared for their virulence. The plants were observed for 3 weeks after inoculation for mortality of seedlings, and observations were recorded from one protray of 96 plants each. The plants were maintained at net-house conditions.

#### 2.3.6. Confocal Scanning Laser Microscopy to Confirm Virulence In Vivo

Fine sections of infected tomato roots were made, and the slides were prepared by using mounting oil (Invitrogen, Carlsbad, CA, USA). Slides were examined under CSLM (Nikon Eclipse 90i) for colonization and spread of *R. solanacearum* NAIMCC-B-01630 in tomato tissues. The X, Y, and Z plane images were taken under excitation of 488 nm laser, and images were acquired by using the NIS element 3.2.3 program (Nikon). The images of the right focal plane were used for colonization study. Grayscale image was visualized under TD channel (it produces transmitted light differential interference contrast images).

### 2.4. Statistical Analysis

Laboratory experiments were carried out in a completely randomized design (CRD) and net-house experiments in a randomized complete block design (RCBD). Data were subjected to analysis of variance, using statistical package for Social Sciences Version 16.0 (SPSS 16.0, IBM, Armonk, NY, USA). Data were compared with Duncan’s Multiple Range Test (DMRT) at *p* ≤ 0.05.

## 3. Results and Discussion

Phenotypic conversion (PC) in *R. solanacearum* is a well-known phenomenon, which causes changes in cell physiology and gives rise to avirulent-type colonies. Since the pure culture of *R. solanacearum* contains the different fraction of cells differing in their virulence, it was observed in our experiments that the reversal of PC and appearance of colonies with higher EPS production occurs due to two physic-chemical factors (nutrient deprivation and *P. emblica* fruit extract), which could cause a reversal of PC to virulent type. The results obtained and discussions on the reversal of PC, and a protocol for maintaining uniform virulent-type colonies in lab conditions are presented here.

### 3.1. Reversal of PC Due to P. emblica Extract and Nutrient Deprivation

**Figure 3:**
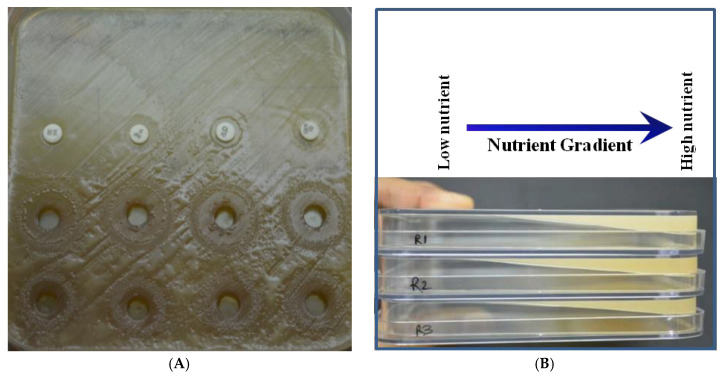

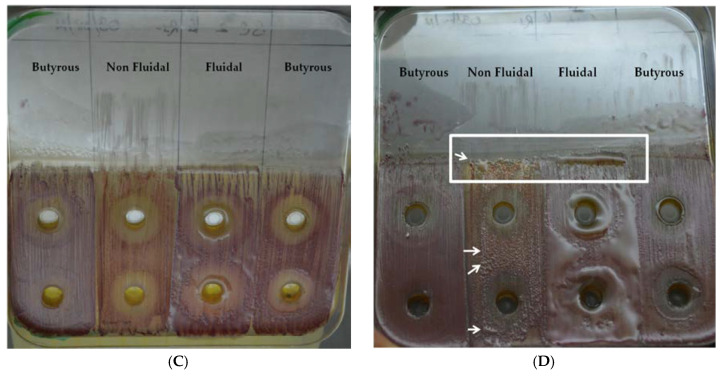
Protocol for induction of virulence colony types in *R. solanacearum*, using full-strength KMTTC medium. (**A**) Induction of the fluidal colony by Indian gooseberry extract. (**B**) Slanting for induced nutrient deprivation in *R. solanacearum*. (**C**) Swabbing of *R. solanacearum* culture differ in colony type in slanting KMTTC agar medium. (**D**) Induction of fluidal colony after three days; white rectangle indicates the effect of nutrient deprivation (slanting edge), whereas white arrows indicate the effect of Indian gooseberry fruit extract; left-to-right sequence = NAIMCC-B-01630 (butyrous), NAIMCC-B-01630 (non-fluidal), NAIMCC-B-01630 (fluidal), and TB-01838 (butyrous).

The reversal of PC was tested in four colony morphotypes (one non-fluidal, one fluidal, and two butyrous), using full-strength KMTTC. Strains showed their original colony morphotypes in the initial 24 h. After three days of incubation, the non-fluidal colonies started converting into fluidal colonies close to agar wells and also toward the edges of the nutrient-depleted area (Table 1; Figure 3). The presence of different virulent forms in the pure culture of *R. solanacearum* has been shown by ion-exchange chromatographic studies [9,10]. The ion-exchange chromatographic approach showed that the so-called single colony is developed from multiple cells associated with each other, differing in their virulence attenuation. The present study also provides evidence that different cell types can be obtained from the same colony, under different culture conditions. The current protocol could be useful in collecting low attenuated cells from the mixture for further studies on virulence and biocontrol. Since the studies on cellular physiology of *R. pseudosolanacearum* indicate that low pH (5.5) could enhance the expression of virulence genes [24], *P. emblica* extract (rich in ascorbic acid) could be able to influence the appearance of virulent-type colonies. Since all the strains of *R. solanacearum* have variable induction rates, providing a gradient of nutrients could be useful.

Similarly, in our protocol, the fluidal colonies emerged where the most-suited nutrient concentration was received. To further confirm, the same experiment was repeated with 1/50 strength KMTTC. Although the non-fluidal and butyrous colonies did not emerge initially, all three colony types gave fluidal colonies after four days of incubation (Table 1). Due to low nutrient availability, cells might have utilized the nutrients present in the extract (after it diffused to a safer concentration). The effects of *P. emblica* extract and nutrient deprivation were found to supplement each other. However, nutrient deprivation alone or *P. emblica* extract alone could generate the fluidal colonies, but better effects were observed when used together.

### 3.2. Bacteriostatic Effect of P. emblica Extract

**Figure 4:**
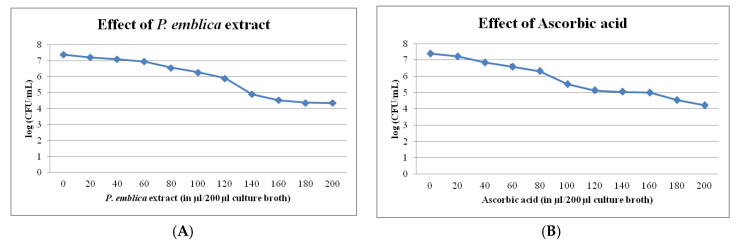
Effect of different concentrations of *P. emblica* extract (**A**) and ascorbic acid (**B**) on the viability of *R. solanacearum* NAIMCC-B-01630 cells in log (CFU/mL).

Since *P. emblica* fruit has medicinal values against different pathogens [25], it may be killing the *R. solanacearum* cells in zones near wells. Once the compounds are diffused to a safer level for *R. solanacearum*, the cells that survive from this killing effect might be able to grow profusely (Supplementary Materials Figure S1). Such cells may also utilize the traces of *P. emblica* extract as nutrient, as this was not observed in liquid cultures where colony growth was stopped, and no resurgence was reported. The direct effect of this extract is sublethal for bacteria, as it was seen from the broth culture study (Figure 4). There are reports of *P. emblica* preparation having antibacterial activities suppressive to the pathogen [26]. Still, its application against *R. solanacearum* has been found to aggravate the pathogen after 2–3 days in our study. This phenomenon can be used for maintaining virulence in *R. solanacearum* strains so that screening of good biocontrol agents can be done.

The suppression zone found to have low pH (Supplementary Materials Figure S2); therefore, it was suspected that a part of *P. emblica* effect might be due to ascorbic acid present in it. The use of ascorbic acid at 10,000 mg/L was found to have similar effects, though the extent was less, which may be due to the presence of other compounds, like tannins in the crude extract [27], that could be adding to the induction. The resurgence occurs after the removal of bacteriostatic pressure, which might be helping the pathogen to rebuild the virulent community, similar to the phenomenon seen by a sublethal dose of antibiotics.

The nature of the suppression by *P. emblica* was studied to check if the effect is bacteriostatic or bactericidal. The *P. emblica* extract suppressed the *R. solanacearum* colonies in a rate correlating to its concentration. However, complete killing of cells was not observed. The CFU in broth without extract was 25 × 10^6^, which declined sharply with increasing concentration of the extract to 80 µL (37 × 10^5^), followed by a slow decline to 41 × 10^4^ with 200 µL extract (Figure 4). This result indicated that, even if the cells are grown at 100% concentration, some of the cells could survive and were able to form colonies later. Similar results were also obtained with 10,000 mg/L ascorbic acid. The CFU in broth without ascorbic acid was 25 × 10^6^, which declined sharply till 40 µL (72 × 10^5^), followed by a slow decline to 21.5 × 10^3^ with 200 µL ascorbic acid. Suppression by different concentrations of *P. emblica* indicates bacteriostatic effect. The cells could develop to fluidal colonies once the bacteriostatic pressure was removed due to the diffusion of compounds.

**Figure 5:**
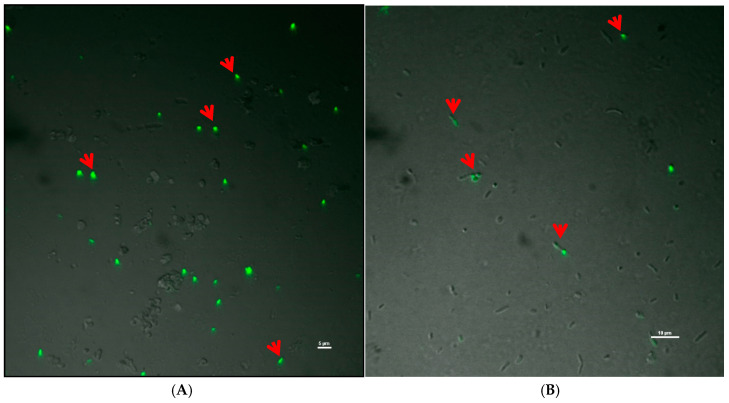
Live–dead cell imaging using SYTO-9 and propidium iodide under confocal scanning laser microscope; arrow indicating live cell signals, (**A**) cells from extract diffusing zone (induced zone), and (**B**) cells from region away from the diffusion zone.

To further confirm, the live–dead cell imaging was performed by taking the biomass from the diffusion and non-diffusion zone. It was found that some of the bacteria were still alive in the diffusion zone, as indicated in red arrows in Figure 5. SYTO-9-stained yellow fluorescing cells could be spotted in the suppression zone, indicating live cells. The *Bac*Light staining proved that the *P. emblica* extract did not kill the *R. solanacearum* population completely around the suppression zone. The live cells present here started growing once the extract started diffusing out. Once the cells survived the challenge of nutrient deprivation/*P. emblica* extract, they may be getting some induction for forming virulent colony type. In the suppression zone, resurgence could also be due to the availability of free space and nutrients. It is also reported that virulence genes of *R. solanacearum* get activated [28] due to the environmental pressure.

### 3.3. Role of Nutrient Deprivation

**Figure 6:**
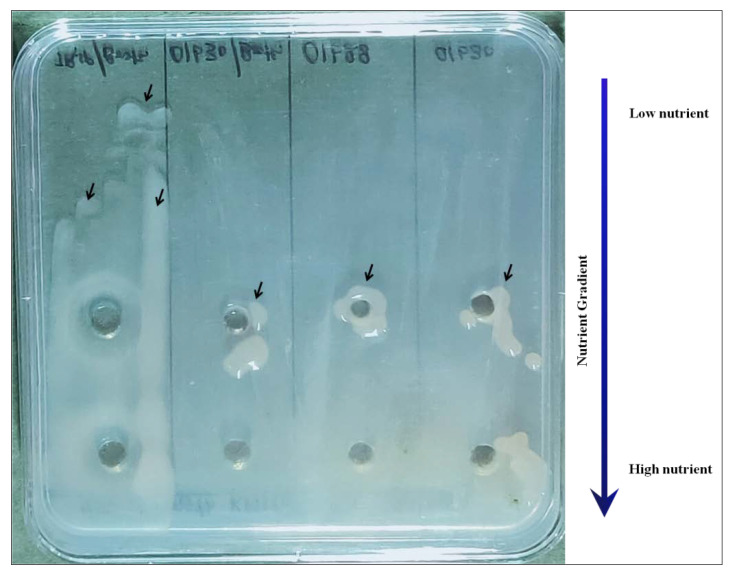
Induction of fluidal colony in 1/50 strength KMTTC media with Indian gooseberry extract in three days incubation; black arrow indicating virulent type colonies; left-to-right sequence = NAIMCC-B-01630 (fluidal), NAIMCC-B-01630 (non-fluidal), TB-01838, and NAIMCC-B-01630 (butyrous).

Nutrient deprivation was found to play synergistic roles in the induction of virulent colony type. We found that the PC has some relationship with nutrient availability. Furthermore, nutrient deprivation studies were made (using 1/50th strength KMTTC) for verifying its effect on the occurrence of colony types in *R. solanacearum* NAIMCC-B-01630 and TB-01838. In slanting plates, nutrients were gradually depleted toward the center of the plate. The emergence of fluidal colonies in Figure 3 indicated the induction by the nutrient deprivation at the edges of the slant, which could be further enhanced by using 1/50th strength (depend on strains) KMTTC. Initially, there was no growth in all the colony types, and induction started after three days of incubation (Figure 6 and Table 1). The full-strength KMTTC converted non-fluidal colonies to fluidal type. Still, no effects were observed on butyrous colonies, whereas 1/50th strength KMTTC causes reversal of all colonies’ types to fluidal. Although the reduction is based on the kind of *Ralstonia* strain, further studies showed similar effects in 12 out of 16 cultures used (data not shown).

Yang et al. [29] reported a change in gene expression of the housekeeping genes in *Ralstonia pseudosolanacearum* CQPS-1, under the influence of temperature, nutrient, and plant-derived hydroxycoumarins. In our study, it was found that the induction in the cells survived in low nutrient availability, and this may contribute to the reversal of PC strains. Since *R. solanacearum* can survive in poor nutrient conditions [30], the surviving cells may get signals for PC reversal. On a similar line, the suppressed cells surrounding the agar wells start emerging as fluidal colonies.

### 3.4. Effect on Other Virulence Factors

**Figure 7:**
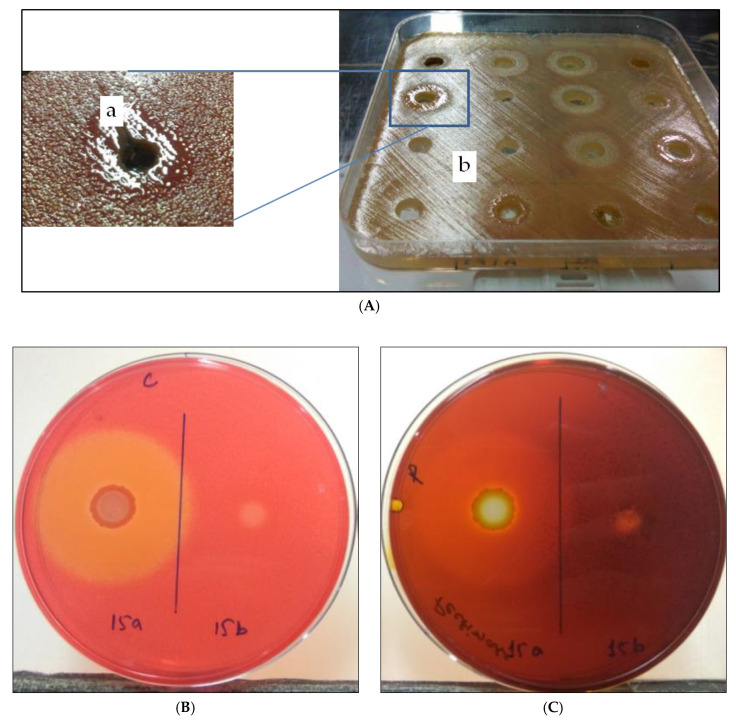
Effect of this protocol on induction of two other virulence factors (production of cellulase and pectinase). (**A**) Induction in appearance of fluidal colonies, (**B**) difference in cellulase production capabilities between induced and non-induced colonies (between zone ‘a’ and ‘b’), and (**C**) difference in pectinase production capabilities between induced and non-induced colonies.

Cellulase and pectinase are two other crucial virulence factors of *R. solanacearum*. A difference in cellulase and pectinase production was observed after induction by this protocol (Figure 7). In the cellulase production assay, the PC reversal strain had a 34 mm zone as compared to 6 mm in the non-reversal strain. Similarly, in the pectinase production assay, a 33 mm zone was observed from the reversal strain against a 6 mm zone from the non-reversal strain. The subculturing of these two cell fractions indicated stability to further generations, as well. Once induced, it may be used for a longer time, without losing these virulence factors. However, variations among the different strains were found. Peeters et al. [31] reported that the cellulase and pectinase enzymes are pathogenicity determinants in *R. solanacearum*. Hence, the phenomenon of PC reversal could be crucial in disease development.

### 3.5. In Vivo Virulence of Converted Fluidal Colonies

**Figure 8:**
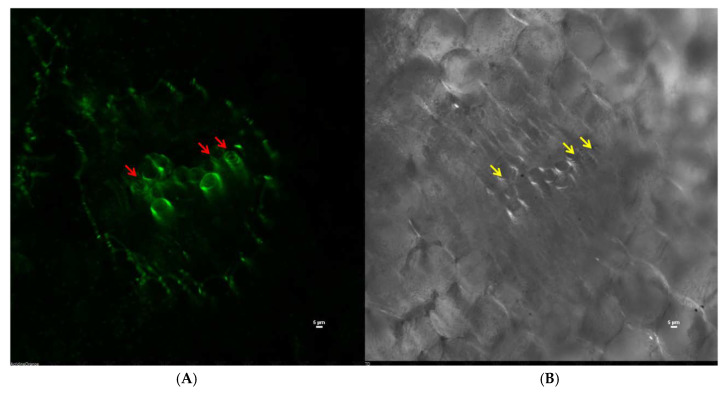

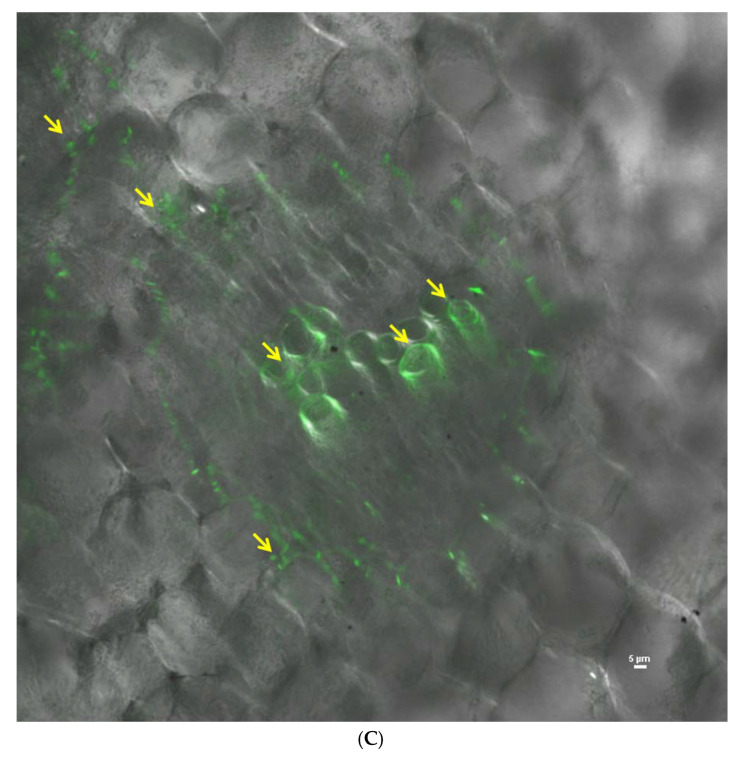
Confocal scanning laser microscope images showing colonization of *gfp*-tagged *R. solanacearum* NAIMCC-B-01630 in tomato root; red arrows indicate vascular colonization of the tagged pathogen in 488 channel, and yellow arrows indicate the same in the TD channel (**A**) vascular colonization as seen in the 488 nm channel, (**B**) vascular colonization as seen in TD channel, and (**C**) superimposed image of 488 and TD channels.

The in vivo trial showed wilting of the plants by the fluidal *R solanacearum* strain. The fluidal strain wilted both susceptible and resistant cultivar to the extent of 77.74% and 24.30%, respectively (Table 2). On the other hand, the non-fluidal strains did not cause any significant disease symptoms in either of the cultivars. In the case of converted fluidal strain, the mortality of 63.89% was reported in the susceptible cultivar, and 14.58% in the resistant one. The enhanced virulence in converted fluidal colonies may be due to the cells with high EPS production and good hydrolytic enzyme activities. The cellulase and pectinase could help the pathogen in gaining entry to vascular bundles, and high EPS production could block the xylem vessels and cause wilting of the plants. CSLM indicated the colonization of green fluorescent protein (*gfp*) transconjugants *R. solanacearum* in the vascular tissues and infected the plants in a week. In Figure 8, arrows indicate vascular colonization of the tagged pathogen. Results showed that the expression of *gfp* did not interfere with the virulence of *R. solanacearum.* Andargie et al. [32] also used *gfp*-transformed *Ustilaginoidea virens* to study the colonization in rice spikelets, and the entry of the pathogen was found to be unaffected by the transformation. During the infection process, *R. solanacearum* uses intercellular spaces to reach the outskirts of the vascular cylinder and colonizes around the stele and further grows and fills xylem vessels [17]. A similar colonization pattern around the stele region and in vascular bundles was observed in this study. The internal colonization of *gfp*-tagged *R. solanacearum* in the present study confirmed that the converted fluidal colony could colonize the plants and cause disease symptoms. In a study using *Clavibacter michiganensis* subsp. *michiganensis* causing bacterial wilt and canker of tomato, *gfp* tagging and expression indicated colonization in vascular bundles and nearby tissues [33].

These findings indicate that the protocol could be useful in inducing virulent colonies. The slanting of the media provided the necessary gradient of nutrient availability of the agar well diffusion with *P. emblica* extract, which would have imparted the required induction for fluidal colonies to emerge. Therefore, putting together both of these factors in one plate could be very much user-friendly in inducing virulent colony types in *R. solanacearum*. The method could be handy for the labs working on *R. solanacearum* and culture collection for its preservation. The phenomenon could also be useful in the research on cross-protection by PC strains. If stresses like nutrient deprivation and bacteriostatic pressure could reverse the applied avirulent strain to virulent ones, it could cause further aggravation of the disease. Therefore, the effect of different environmental stresses should be taken into consideration before recommending PC strains for cross-protection.

## 4. Conclusions

Sudden shift and loss of virulence (PC) in *R. solanacearum* has been reported and is a critical issue in its pathogenicity experiments in general and biocontrol research in particular. There was a need for a protocol to maintain its virulence for an extended period for uniform experimentation. *P. emblica* fruit extract, coupled with nutrient deprivation, could cause a reversal of PC to fluidal type. Both of the factors can be synergistically employed for getting virulent colony types in *R. solanacearum* for research on its biocontrol. However, the reversal of PC could be a serious concern when PC strains are used as biocontrol agents as cross-protection against virulent *R. solanacearum* strains in the natural ecosystem.

## Figures and Tables

**Table 1:** Effect of the given protocol on the conversion of colony morphotypes.

SN. Colony Morphology (before Experiment)		Colony Morphology around Wells Containing *Phyllanthus emblica* Extract			
		Full-Strength KMTTC		1/50 Strength KMTTC	
		24 h	72 h	24 h	72 h
1	Butyrous (NAIMCC-B-01630)	NF	NF	NG	F
2	Butyrous (TB-01838)	NF	NF	NG	F
3	Non-fluidal (NAIMCC-B-01630)	NF	F	NG	F
4	Fluidal (NAIMCC-B-01630)	F	F	NG	F

Note: NF = non-fluidal, F = fluidal, NG = no growth.

**Table 2:** Mortality in the tomato plants due to inoculation different physiological states of *R. solanacearum*.

S. No.	Physiological State of *R. solanacearum*	Mortality (%)	
		Susceptible Cultivar (Pusa Ruby)	Resistant Cultivar (Arka Rakshak)
1	Control	0.00 d	0.00 c
2	Fluidal	77.74 a	24.30 a
3	Non-fluidal	11.11 c	1.39 c
4	Converted fluidal	63.89 b	14.58 b

Notes: data analyzed in a randomized complete block design (RCBD); the means were separated by DMRT at *p* ≤ 0.05. Values with the same alphabet are not significantly different.

## Supplementary Materials

The following are available online at https://www.mdpi.com/1660-4601/17/12/4274/s1. Figure S1: Application, suppression, and resurgence observed after *P. emblica* fruit extract application. Figure S2: pH of the medium and bacteriostatic zone.
