# Supplementary material for: A Simplified Protocol for Reversing Phenotypic Conversion of Ralstonia solanacearum during Experimentation
Source: Int J Environ Res Public Health. 2020 Jun 15;17(12):4274. doi: 10.3390/ijerph17124274 (PMC7344456; doi:10.3390/ijerph17124274)
Supplement: Supplementary file 1 [file ijerph-17-04274-s001.pdf]

International Journal of
Environmental Research and Public Health

MDPI

Supplementary

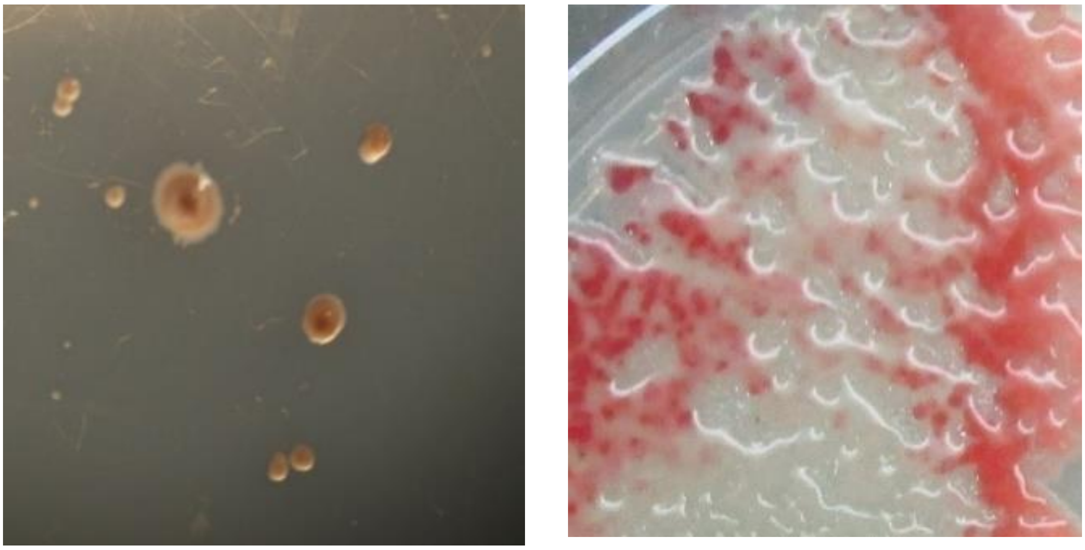

(**A**) (**B**)

Figure S1. Colony morphology of strain used for *gfp* labeling, (**A**) Single colony morphology in stereo microscope, and (**B**) Colonies in Petri plate.

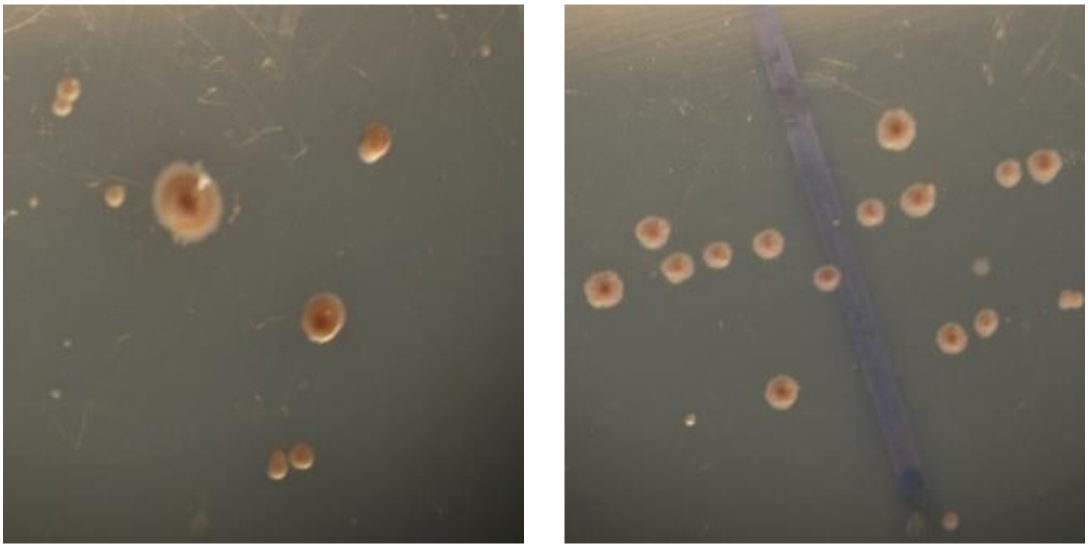

(**A**) (**B**)

Figure S2. Colony morphology of strain before (**A**) and after *gfp* labeling (**B**).
